# First Description of the Role of the Relationship Between Serum Amyloid P Components and Nuclear Factors/Pro-Cytokines During Critical Periods of Toxoplasmic Encephalitis

**DOI:** 10.3390/brainsci14121298

**Published:** 2024-12-23

**Authors:** Gungor Cagdas Dincel, Hasan Tarik Atmaca, Saeed El-Ashram

**Affiliations:** 1 Department of Medical Pathology, Faculty of Medicine, Ankara Medipol University, Ankara 06050, Turkey; 2 College of Life Science and Engineering, Foshan University, 18 Jiangwan Street, Foshan 528231, China; 3 Department of Pathology, Faculty of Veterinary Medicine, Balikesir University, Balikesir 10145, Turkey; 4 Zoology Department, Faculty of Science, Kafrelsheikh University, Kafr El-Sheikh 33516, Egypt

**Keywords:** interleukin-1 beta, nuclear factor kappa B, serum amyloid P, *Toxoplasma gondii*, toxoplasmic encephalitis, tumor necrosis factor-alpha

## Abstract

**Background/Objectives:** *Toxoplasma gondii* (*T. gondii*), an obligate food-borne intracellular parasite, causes severe neuropathology by establishing a persistent infection in the host brain. We have previously shown that *T. gondii* infection induces severe neuropathology in the brain manifested by increased nitric oxide production, oxidative stress, glial activation/BBB damage, increased pro-inflammatory cytokine glia maturation factor-beta and induced apoptosis. **Methods:** The aim of this experimental study was to investigate the serum amyloid P (SAP) components, nuclear factor kappa B (NF-κB), interleukin-1 beta (IL-1β), caspase 1 (Casp 1), tumor necrosis factor-alpha (TNF-α) and complement 3 (C3) gene expressions on the 10th, 20th and 30th days after infection with *T. gondii* in the neuroimmunopathogenesis of toxoplasmic encephalitis (TE) in mouse brains by real-time quantitative polymerase chain reaction. The study also aimed to determine whether there was a correlation between the markers included in the study on these critical days, which had not previously been investigated. The mRNA expression levels of SAP components, NF-κB, IL-1β, Casp 1, TNF-α and C3 were examined. **Results:** The most notable outcome of this investigation was the observation that SAP components exhibited a 13.9-fold increase on day 10 post-infection, followed by a rapid decline in the subsequent periods. In addition, IL-1β expression increased 20-fold, while SAP components decreased 13-fold on day 20 after infection. Additionally, the TNF-α, Casp 1 and NF-κB expression levels were consistently elevated to above normal levels at each time point. **Conclusions:** This study identified SAP components, NF-κB, IL-1β, Casp 1 and TNF-α expressions as playing critical roles in TE neuroimmunopathogenesis. Furthermore, to the best of our knowledge, this is the first study to investigate SAP components during the transition from acute systemic infection to early/medium chronic and chronic infection and to explore the relationship between SAP components and other nuclear factors/pro-cytokines.

## 1. Introduction

*Toxoplasma gondii* (*T. gondii*), an intracellular protozoan parasite capable of infecting all warm-blooded animals, enters the human and animal hosts through the consumption of undercooked or raw meat or by ingesting food or water contaminated with oocysts shed by infected cats [1,2]. *T. gondii* tachyzoites, which multiply rapidly during acute systemic infection, undergo transformation into bradyzoites and form cysts in the early stages of chronic infection after traversing the blood–brain barrier (BBB) and undergoing a series of genetic changes [3,4]. As the infection progresses and reaches the mid-chronic stage, both innate and adaptive peripheral immune cells must continuously maintain the balance between pro- and anti-inflammatory cytokine production [5,6]. During these stages, neuroinflammation, vascular damage and BBB compromise can contribute to neuropathology in both the morphology and function of infected and uninfected neurons and glial cells [5,7,8]. The host immune response and neuroimmunopathogenesis during the transition from systemic acute infection to the early and mid-chronic periods have not been fully investigated and elucidated.

Serum amyloid P (SAP) is a member of the pentraxins, characterized by calcium-dependent ligand binding with a pentameric structure, and is an important component of the innate immune system. Pentraxins play a pivotal role in the binding of microbial pathogens in instances of infection or inflammation, as well as in the recognition and opsonization of cellular debris in pathological conditions [9]. In fatal neurodegenerative diseases such as Alzheimer’s disease, Creutzfeldt-Jakob disease, Pick’s disease, Parkinson’s disease and Lewy body disease, the concentration of SAP components in the brain has been found to increase significantly. SAP components have been shown to play an active role in neurodegeneration [10,11]. However, the role of SAP components in the neuropathogenesis of toxoplasmic encephalitis (TE) during the transition phase from acute to chronic after infection with *T. gondii* and in the chronic phase, including tissue cysts, is still unknown.

Nuclear factor-kappa B (NF-κB), maintained in a latent form in the cytoplasm, is a transcription factor that plays a role in processes such as cell survival and proliferation during inflammation. When stimuli activating NF-κB are present in the environment, the nuclear translocation of NF-κB and activation of gene transcription occur [12]. Interleukin-1 beta (IL-1β) has been shown to play a leading role in triggering signaling associated with NF-κB stimulation in cells lining the walls of the vascular system of the central nervous system (CNS) [13]. In amyotrophic lateral sclerosis (ALS), a mechanism involving tumor necrosis factor-alpha (TNF-α) and the NF-κB pathway, has been shown to cause motor neuron necrosis [14], and there is also evidence that it causes damage to the blood–brain barrier (BBB) [15]. These studies emphasize that NF-κB has important functions in the neuropathogenesis of diseases, despite its physiological roles that cannot be ignored.

IL-1β [16], a pro-inflammatory cytokine expressed at very low levels in the CNS, is produced as a biologically inactive pro-form (proIL-1β) and is generated by the cleavage of this inactive proIL-1β by caspase-1 (Casp 1), forming the so-called inflammasome [17,18]. Severe neuropathologies resulting from the overexpression of IL-1β have been demonstrated in an ischemic stroke model [19]. Furthermore, pro-inflammatory cytokines such as IL-1β and TNF-α have been implicated in the pathogenesis of neurodegenerative diseases such as experimental autoimmune encephalomyelitis (EAE), multiple sclerosis (MS) and PD [20,21,22]. Therefore, both in vivo and in vitro studies strongly suggest that IL-1β and TNF-α play a major role in the neuropathogenesis of neuronal degeneration, which is significantly increased in glial cells under neuroinflammatory and neurodegenerative conditions.

While there are studies on the neuropathogenesis of TE, the neuroimmunological mechanisms remain incompletely elucidated. Furthermore, to our knowledge, SAP components have not been investigated during the transition from acute systemic infection to early/mid-chronic and chronic infection, and there are no studies on the relationship between SAP components and other nuclear factors/pro-cytokines. The aim of this study was to investigate the association of SAP components, NF-κB, IL-1β, Casp 1, TNF-α and C3 expression with TE-associated neuropathology on day 10 (transition from acute to early chronic phase), day 20 (transition from early/middle chronic phase) and day 30 (chronic phase) post-infection, which is considered to be the transition phase from systemic acute infection to early chronic phase, and to show whether there is a day-dependent correlation between them.

## 2. Materials and Methods

### 2.1. Ethics Statement

This research was conducted in full compliance with the guidelines set forth by the National Centre for the Replacement, Refinement, and Reduction of Animals in Research (NC3Rs). All experimental protocols and animal handling procedures were approved by the Animal Care Committee of the University of Kırıkkale (Permit Number: 11/02-11/-2 November 2011).

### 2.2. Animal Infection Model and Experimental Procedures

The study utilized Swiss albino female mice, aged 12–16 weeks and weighing 22–25 g, which were sourced from the Private Experimental Animal Production Laboratory (Ankara, Turkey). These animals were continuously monitored for survival, clinical condition and body weight throughout the study, up until tissue collection. The low-virulence ME-49 strain of *T. gondii* was used for infection, as outlined by Atmaca et al. (2014) [23]. *T. gondii* tissue cysts were collected from the brains of mice that had been intraperitoneally inoculated with 20 tissue cysts. For the experimental infection, the mice were intraperitoneally administered 15 ME-49 tissue cysts suspended in 0.25 mL of sterile physiological saline. Post-inoculation, no typical symptoms of acute toxoplasmosis, such as lethargy, ruffled fur or hunched posture, were observed in the infected mice. The mice were anesthetized with pentobarbital (10 mg/kg) and euthanized by cervical dislocation. Six mice from each of the 10th, 20th and 30th days post-inoculation were sacrificed for tissue collection. Additionally, six healthy control mice, sacrificed at the start of the study, were used for total RNA extraction and cDNA synthesis.

### 2.3. Necropsy and Rapid Determination of Tissue Cysts

The brain tissues were rapidly excised within one minute following the euthanasia of the animals to ensure preservation of the cellular integrity. The detection of *T. gondii* tissue cysts was conducted using the squash smear cyst detection technique, originally developed and reinterpreted by Dincel (2017) [24]. In this method, five small fragments of fresh brain tissue were randomly selected from the infected group and placed on poly-L-lysine-coated glass slides. These tissue pieces were initially fixed in 10% neutral-buffered formaldehyde for one minute, followed by rehydration through a graded ethanol series for 45 s. The sections were then stained with Mayer’s hematoxylin for 35 s, followed by eosin for 45 s. After staining, the slides were rinsed with distilled water, dehydrated in a graded alcohol series for 1 min, cleared in xylene for 3 min and finally mounted on glass slides.

Both unstained and H&E-stained sections were examined using light microscopy (Olympus, Tokyo, Japan). The analysis of the tissue samples obtained through the squash smear technique was performed in two distinct stages: first by evaluating the unstained preparations to observe natural morphological features, followed by examination of the H&E-stained sections to assess detailed structural and pathological changes. This dual approach provided a comprehensive evaluation of *T. gondii* cysts in the brain tissue.

### 2.4. Histopathological Examination

Brain tissue samples were preserved in 10% neutral-buffered formaldehyde for 48 h, followed by a wash with tap water overnight. The tissues were then processed through standard dehydration steps using graded alcohol and xylene, before being embedded in paraffin. Serial sections of paraffin-embedded tissue were cut to a thickness of 4–5 μm and mounted on glass slides. The brain sections were further sliced to 5 μm, stained with hematoxylin and eosin (H&E) and examined using a light microscope (Olympus BX51 equipped with a DP25 camera, Tokyo, Japan). This study focused on analyzing sagittal brain cross-sections from both healthy control and infected groups.

### 2.5. Immunoperoxidase Examination

To detect *T. gondii* antigens, immunohistochemistry was conducted using an indirect streptavidin/biotin immunoperoxidase method (HRP; Thermo Scientific, Waltham, MA, USA) on 4–5-μm-thick paraffin-embedded tissue sections. A polyclonal antibody specific to *T. gondii* was utilized. The procedure followed the protocol outlined by Dincel and Atmaca (2015) [25]. Tissue sections were mounted on adhesive slides, deparaffinized through three 5-min xylene treatments and rehydrated with a graded series of ethanol and distilled water. Antigen retrieval was performed by heating the slides in citrate buffer (pH 6.0; Thermo Scientific) for 20 min. Endogenous peroxidase activity was blocked by incubating sections in 3% hydrogen peroxide in absolute methanol for 7 min at room temperature. Between steps, sections were washed three times in phosphate-buffered saline (PBS, pH 7.4) for 5 min. To minimize non-specific binding, a blocking serum was applied for 5 min. Subsequently, the sections were incubated with the polyclonal *T. gondii* antibody in a humidified chamber at room temperature for 1 h, followed by 15-min incubations with a biotinylated secondary antibody and streptavidin–peroxidase, respectively. Diaminobenzidine (DAB) chromogen was applied for 5–10 min to develop the color reaction, and Mayer’s hematoxylin served as a counterstain for 1–2 min. DAB staining was controlled under a microscope for 10 min to ensure precision. The slides were analyzed immediately after preparation, with sections viewed and photographed using a binocular light microscope (Olympus BX51 equipped with a DP25 camera, Japan) at 20× magnification. For negative controls, the primary antibody step was omitted to confirm the absence of non-specific endogenous peroxidase or biotin activity.

### 2.6. Total RNA Extraction and cDNA Synthesis

Approximately 20 mg of tissue samples were preserved using RNA Stabilization Reagent (RNAlater, Qiagen, Hilden, Germany) and subsequently homogenized with the TissueLyser II (liver and muscle: 2 cycles of 2 min; other tissues: 2 cycles of 5 min; Qiagen). Total RNA extraction was performed with the RNeasy Mini Kit (Qiagen) following the manufacturer’s protocol on the QiaCube system (Qiagen). The isolated RNA was then reverse-transcribed into complementary DNA (cDNA) using the High-Capacity cDNA Reverse Transcription Kit (Thermo Fisher Scientific, Waltham, MA, USA). A 10-µL RNA sample was combined with 2 µL of 10× RT Buffer, 0.8 µL of 25× dNTP mix, 2 µL of 10× RT Random Primers, 1 µL of MultiScribe Reverse Transcriptase and 4.2 µL of DEPC-treated water. The reverse transcription process was conducted at 25 °C for 10 min, followed by incubation at 37 °C for 120 min and a final step at 85 °C for 5 min using the Veriti 96-Well Thermal Cycler (Applied Biosystems, Foster City, CA, USA). The concentration and quality of the resulting cDNA were evaluated and quantified using the Epoch Spectrophotometer System with a Take3 Plate (Biotek, Winooski, VT, USA).

### 2.7. Relative Quantification of Gene Expression

Relative C3, NF-κB, IL-1β, Casp 1, TNF-α and SAP expression was assessed by real-time quantitative PCR by using the cDNA synthesized from brain RNA in a StepOne Plus Real-Time PCR System (Applied Biosystems, Foster City, CA, USA) with the FastStart Universal SYBR Green Master Kit (Roche Diagnostics, Basel, Switzerland) according to the manufacturer’s instructions. In brief, 1 μL specific primer (0.5 μL forward primer and 0.5 μL reverse primer), 25 μL of SYBR Green QPCR Master Mix (Bio-Rad, Hercules, CA, USA), 5 μL diluted cDNA and 19 μL RNase-free water were mixed. The sample was denatured at 95 °C for 10 min, followed by 40 cycles of 95 °C for 10 s and at 60 °C for 1 min. PCR products labeled with SYBR green were detected. The sequences of the specific primers are shown in Table 1 (Primer Design Ltd., Southampton, UK).

Reference sequences for the SAP components, NF-κB, IL-1β, Caspase 1, TNF-α and C3 were sourced from Wilcockson et al. (2002) [26]. The results were quantified as relative fold changes and compared against the control groups. Expression of β-actin served as the endogenous control across all cell groups. Each target assay was conducted in triplicate on a 96-well optical plate, with reaction mixtures consisting of 9 µL cDNA (100 ng), 1 µL Primer Perfect Probe mix and 10 µL QuantiTect Probe PCR Master Mix (Qiagen, Hilden, Germany), totaling 20 µL per well. The thermal cycling protocol included an initial incubation at 50 °C for 2 min, followed by 95 °C for 10 min and then 40 cycles of 94 °C for 15 s and 60 °C for 1 min [27]. Expression data were analyzed using the 2^−ΔΔCt^ method [28], presented as fold changes relative to other experimental animal groups.

### 2.8. Statistical Analysis

The statistical analysis of the mRNA levels for the SAP components, NF-κB, IL-1β, Casp 1, TNF-α and C3 was conducted using one-way analysis of variance (ANOVA) followed by Tukey’s multiple comparison test. Data are expressed as the means ± standard deviation (SD). All statistical evaluations and graphical representations were generated using GraphPad Prism version 7.0 (GraphPad Software, La Jolla, CA, USA). A *p*-value of <0.05 was considered indicative of statistical significance.

## 3. Results

### 3.1. Histopathological Findings

**Figure 1:**
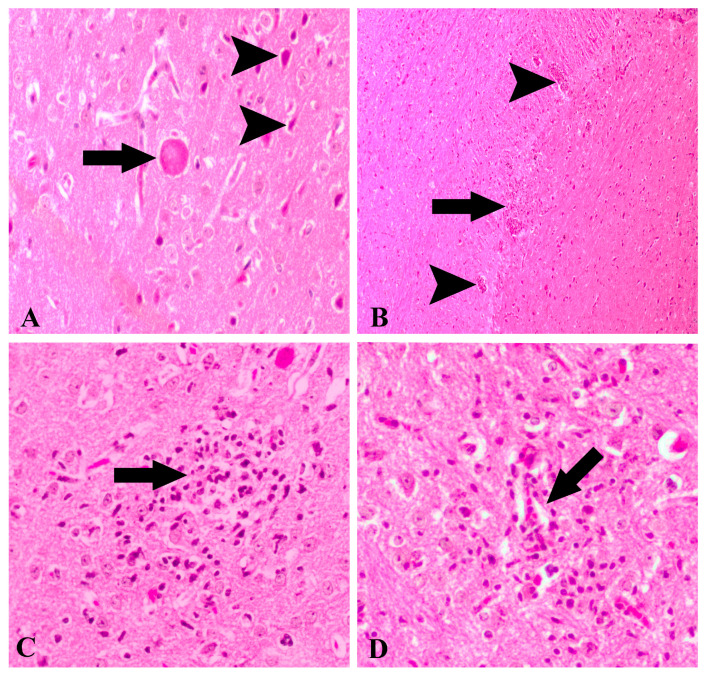
Histopathology of toxoplasmic encephalitis stained by hematoxylin and eosin. (**A**) *Toxoplasma gondii* tissue cysts (arrow). Necrotic/degenerative neuronal cells (arrowheads). The 10th day of infection, 20× magnification. (**B**) Severe perivascular mononuclear cell infiltration (arrowheads) and gliosis focus (arrow). The 20th day of infection, 10× magnification. (**C**) Glia proliferation (arrow) in the brain. The 30th day of infection, 20× magnification. (**D**) Glia proliferation and perivascular mononuclear cell infiltration (arrow). The 30th day of infection, 20× magnification.

H&E-stained brain sections from healthy control animals showed normal architecture, but increasing neurohistopathology was observed in the 10th, 20th and 30th day post-infection groups. Notable histopathological findings included non-purulent and/or necrotizing meningitis, neuronal degeneration, tissue cyst (Figure 1A) and shrunken Purkinje cells/neurons with intense eosinophilic and necrotic appearance (Figure 1A) and focal gliosis (Figure 1B,C). Furthermore, hyperemia, perivascular mononuclear cell infiltration (Figure 1B–D) and glia proliferation (Figure 1B–D) were observed throughout the brain. Differences in neurohistopathological findings were observed at the 10th, 20th and 30th days after infection with *T. gondii*. The most striking neurohistopathological evaluation was that the findings observed on the 10th day after infection with *T. gondii* were at a milder level compared to the 30th day after infection groups.

### 3.2. Immunoperoxidase Findings

**Figure 2:**
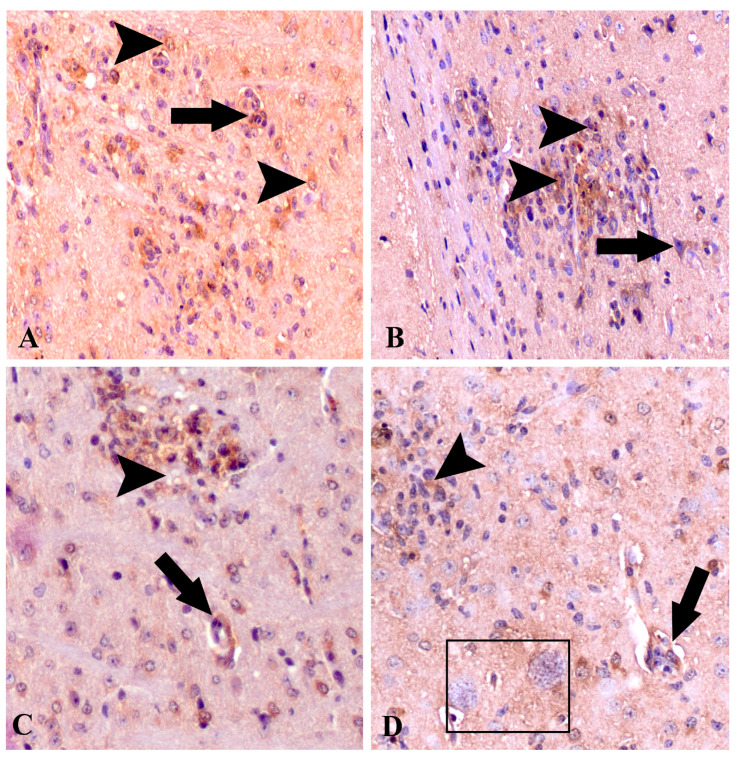
Immunohistochemical stain for *Toxoplasma gondii* in the brain. (**A**) Immunopositivity for *Toxoplasma gondii* antigens in neurons (arrowheads) and glial cells (arrows). The 10th day of infection, 20× magnification. (**B**) Immunopositivity for *Toxoplasma gondii* antigens in necrotic neurons (arrow) and glial cells (arrowheads). The 20th day of infection, 20× magnification. (**C**) Immunopositivity for *Toxoplasma gondii* antigens in the glial proliferation area (arrowhead) and vessel endothelial cells (arrow). The 20th day of infection, 20× magnification. (**D**) Immunopositivity for *Toxoplasma gondii* antigens in the glial proliferation area (arrowhead) and vessel endothelial cells (arrow). The black rectangle highlights two distinct tissue cysts, each enclosed within the rectangle. The 30th day of infection, 20× magnification.

Anti-*Toxoplasma gondii* immunopositivity was seen particularly in neurons and endothelial cells, which was supported by histopathological findings (Figure 2A). Immunoreactivity in necrotic neurons and areas characterized by glial proliferation and perivascular mononuclear cell infiltration was also an important finding (Figure 2B–D). Immunopositivity was seen in the glial cells that surround the tissue cyst (Figure 2D).

### 3.3. mRNA Gene Expression Findings

#### 3.3.1. SAP mRNA Expression

**Figure 3:**
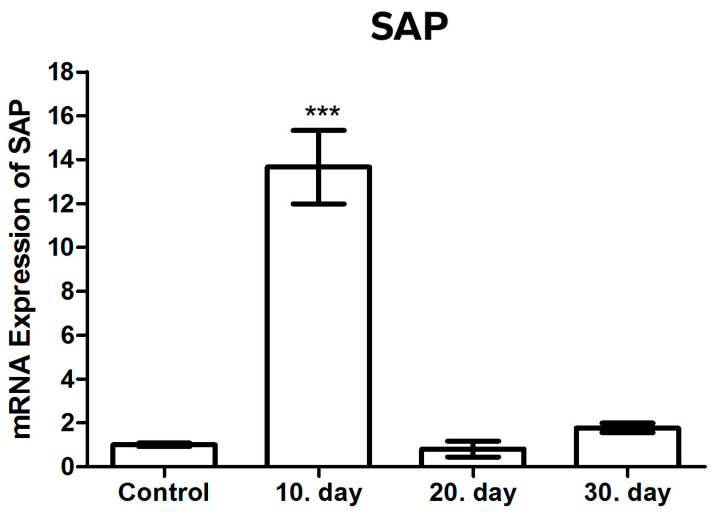
mRNA expression levels of SAP in toxoplasmic encephalitis. (*** *p* < 0.001).

The expression levels of SAP mRNA were analyzed using quantitative reverse transcription-polymerase chain reaction (qRT-PCR), with β-actin used as a normalizing control and presented as a fold change. Statistical significance was indicated by the use of asterisks (*** *p* < 0.001). The expression of SAP mRNA was observed to increase by a factor of 13.9 on day 10 following infection with *T. gondii* in comparison to the healthy control group. This increase was found to be statistically significant (*p* < 0.001) (Figure 3 and Table 2).

It is noteworthy that the highest levels of SAP mRNA in comparison to the healthy control group were observed only on post-infection day 10, in contrast to the other infected groups (post-infection days 20 and 30).

A rapid decrease in SAP mRNA levels was observed on the 20th and 30th days following infection, reaching a level comparable to that of the healthy control group. Consequently, a statistically significant decrease in the SAP mRNA levels between the 10th day after infection and the 20th and 30th days after infection was another noteworthy finding (*p* < 0.001).

**Figure 4:**
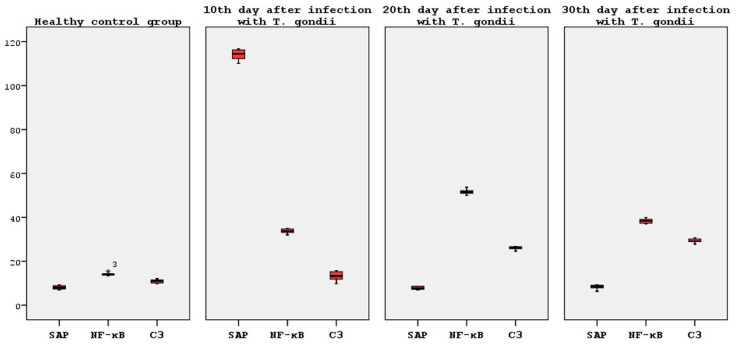
Statistical analysis of the SAP, NF-kB and C3 mRNA expression levels.

No statistically significant result was found in the SAP mRNA levels between the healthy control group and the 20th and 30th days after infection, nor between the 20th and 30th days themselves (*p* > 0.05).The fact that the SAP mRNA levels increased so much on the 10th day, which is the most critical period of infection, and then decreased at the same rate represents a significant finding that cannot be ignored (Figure 4).

#### 3.3.2. NF-κB mRNA Expression

**Figure 5:**
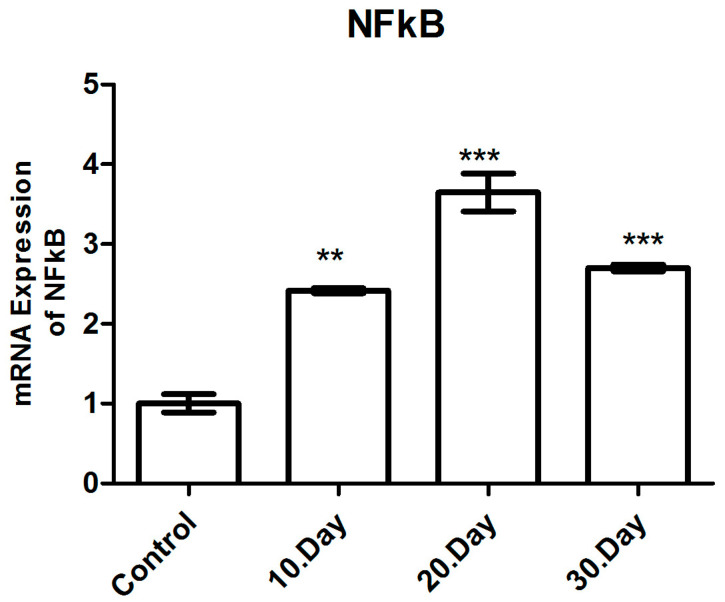
mRNA expression levels of NF-κB in toxoplasmic encephalitis (** *p* < 0.05, *** *p* < 0.005).

The NF-κB mRNA expression levels were analyzed using qRT-PCR, with β-actin used as an internal control, and presented as a fold change. Statistical significance was indicated by the use of asterisks (** *p* < 0.05 and *** *p* < 0.005). The NF-κB mRNA levels were observed to increase by 2.4 (*p* < 0.05), 3.6 (*p* < 0.005) and 2.7 (*p* < 0.005)-fold. On the 10th, 20th and 30th days following infection with *T. gondii*, respectively, compared to the healthy control group, there was a statistically significant increase in the NF-κB mRNA levels in all infected groups (Figure 5 and Table 2).

It is a notable finding that the highest NF-κB mRNA level was observed on the 20th day following infection. A comparison of the post-infection day 20 and post-infection day 10 groups revealed a statistically significant increase in the NF-κB mRNA levels on post-infection day 20 (*p* < 0.05). Although there was a reduction in the NF-κB mRNA levels on post-infection day 30, these levels approached those observed on post-infection day 10 and remained elevated in comparison to the healthy control group. A further noteworthy finding was the statistically significant decrease in NF-κB mRNA levels observed in the day 30 post-infection group when compared to the day 20 post-infection group (*p* < 0.05). Furthermore, no significant difference was observed in the NF-κB mRNA levels between post-infection days 10 and 30 (*p* > 0.05) (Figure 4).

#### 3.3.3. C3 mRNA Expression

**Figure 6:**
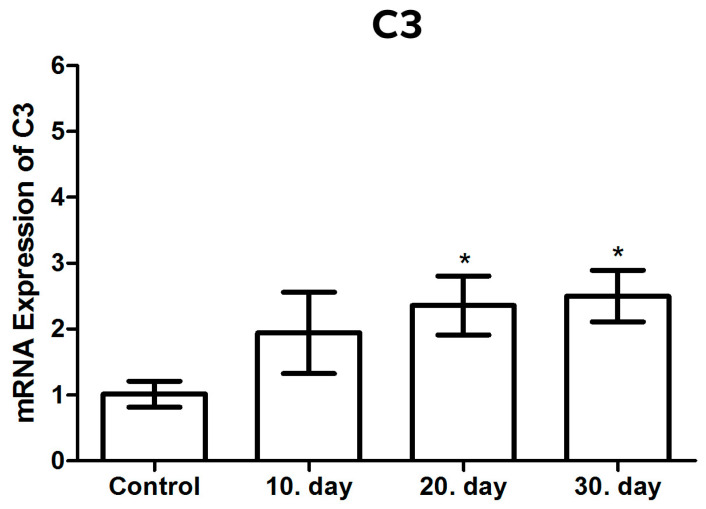
mRNA expression levels of C3 in toxoplasmic encephalitis. * *p* < 0.05.

The C3 mRNA expression levels were analyzed using qRT-PCR, with β-actin used as the normalizing control and presented as a fold change. Statistical significance was indicated by the use of asterisks (* *p* < 0.05). The mean C3 mRNA levels were observed to increase by 2.2 and 2.4-fold (*p* < 0.05) on the 20th and 30th days following infection with *T. gondii*, respectively, in comparison to the healthy control groups (Figure 6 and Table 2).

The highest levels of C3 mRNA were observed on the 20th and 30th days following infection. Although an increase in C3 mRNA levels was also observed on the 10th post-infection day in comparison to the healthy control groups, no statistically significant result was obtained (*p* > 0.05). Furthermore, when the C3 mRNA levels of all the groups (post-infection days 10, 20 and 30) were compared among themselves, no statistically significant result was detected (*p* > 0.05) (Figure 4).

#### 3.3.4. IL-1β mRNA Expression

**Figure 7:**
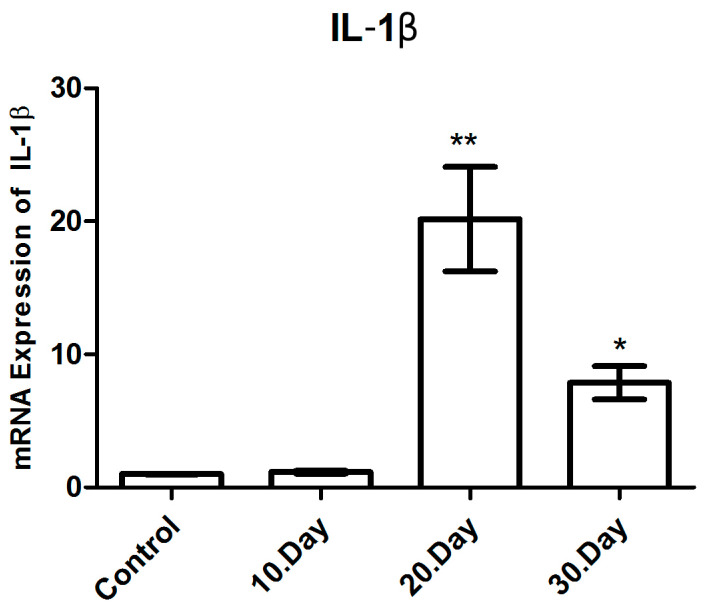
mRNA expression levels of IL-1β in toxoplasmic encephalitis. * *p* < 0.05, ** *p* < 0.005.

The expression levels of IL-1β mRNA were analyzed using qRT-PCR, with β-actin used as an internal control and presented as a fold change. Statistical significance was indicated by the use of asterisks (* *p* < 0.05 and ** *p* < 0.005). A comparison of the day 20 post-infection group with the healthy control group and the day 10 post-infection group revealed that the IL-1β mRNA levels of the day 20 post-infection group exhibited an average 19.8-fold increase (*p* < 0.005) (Figure 7 and Table 3).

It is a notable finding that the highest levels of IL-1β mRNA were observed on the 20th day following infection. Furthermore, a comparison between the post-infection day 10 and healthy control groups with the post-infection day 30 group revealed that the IL-1β mRNA levels in the latter exhibited an average 8.6-fold increase (*p* < 0.05). Despite a reduction in the IL-1β mRNA levels on post-infection day 30, these remained elevated in comparison to the healthy control groups.

**Figure 8:**
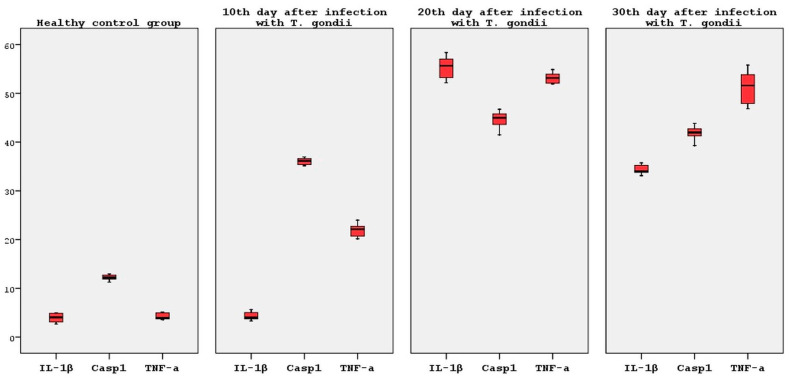
Statistical analysis of IL-1β, Casp1 and TNF-α expression.

A comparison of the IL-1β mRNA levels between the day 20 and day 30 post-infection groups revealed a statistically significant decrease in the IL-1β mRNA levels at day 30 post-infection (*p* < 0.05), which constituted another noteworthy finding. In contrast, no statistically significant result was found when the IL-1β mRNA levels of the healthy control group and the post-infection day 10 group were compared (*p* > 0.05) (Figure 8).

#### 3.3.5. Casp 1 mRNA Expression

**Figure 9:**
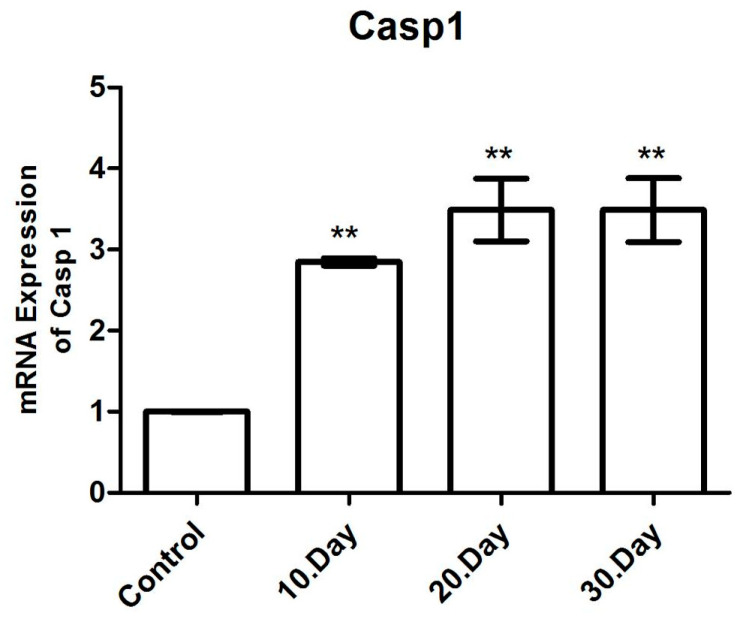
mRNA expression levels of Casp1 in toxoplasmic encephalitis. ** *p* < 0.005.

The Caspase 1 mRNA expression levels were analyzed using qRT-PCR, with β-actin used as the normalizing control and presented as a fold change. Statistical significance was indicated by the use of asterisks (** *p* < 0.005). The results demonstrated that the Casp 1 mRNA levels exhibited a statistically significant increase of 2.9, 3.4 and 3.5-fold on average (*p* < 0.05) on the 10th, 20th and 30th days following infection with *T. gondii*, respectively, when compared to the healthy control groups (Figure 9 and Table 3). The highest Casp 1 mRNA levels were observed on the 20th and 30th days following infection.

No statistically significant difference was detected when the Casp 1 mRNA levels of all the groups (10, 20 and 30 days after infection) were compared to one another (*p* > 0.05) (Figure 8).

No significant increase or decrease was observed in any of the infection groups after infection (Figure 7).

#### 3.3.6. TNF-α mRNA Expression

**Figure 10:**
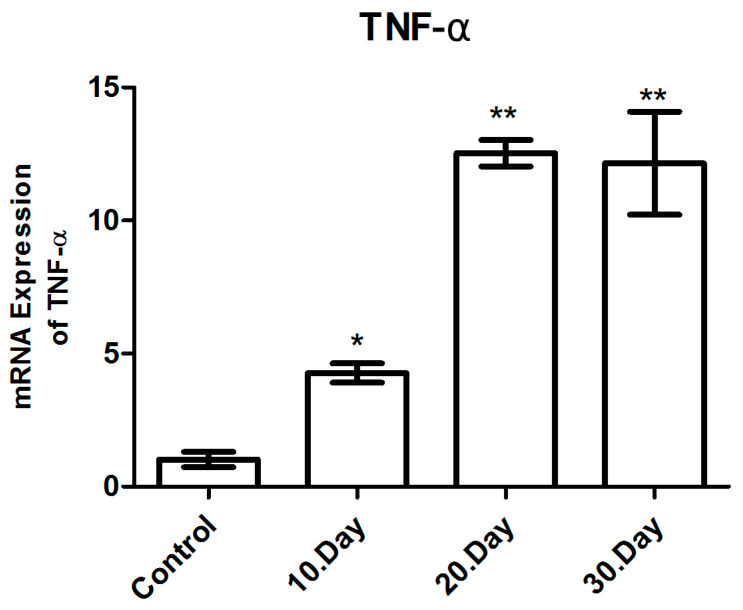
mRNA expression levels of TNF-α in toxoplasmic encephalitis. * *p* < 0.05 ** *p* < 0.005.

The TNF-α mRNA expression levels were analyzed using qRT-PCR, with β-actin used as the normalizing control and presented as a fold change. Statistical significance was indicated by the use of asterisks (* *p* < 0.05 and ** *p* < 0.005). The TNF-α mRNA levels were observed to increase by 4.8 (*p* < 0.05), 12.4 (*p* < 0.001) and 12.1 (*p* < 0.001)-fold on average at days 10, 20 and 30 post-infection with *T. gondii*, respectively, in comparison to the healthy control groups (Figure 10 and Table 3).

It is a notable finding that the highest levels of TNF-α mRNA were observed on the 20th and 30th days following infection. A statistically significant increase in the TNF-α mRNA levels was observed on the 20th and 30th post-infection days compared to the 10th post-infection day (*p* < 0.05). However, when the 20th and 30th post-infection days were compared to one another, it was determined that there was no statistically significant difference in the TNF-α mRNA levels, which were found to be at similar levels (*p* > 0.05) (Figure 8).

## 4. Discussion

While infection of an immunocompetent host with *T. gondii*, the causative agent of toxoplasmosis, is usually asymptomatic, encephalitis with high mortality can be observed in immunocompromised patients, such as cancer and AIDS patients. Severe systemic infections characterized by stillbirth and congenital defects can occur in congenital infections [29,30,31]. We have previously demonstrated at the molecular level that the neuropathogenesis of TE is not solely due to tachyzoites and bradyzoites in the acute and early/late chronic phases. It is also attributed to *T. gondii*-mediated increased pro-inflammatory cytokines (GMF-b, OS and NO secretion at the pathological level) and the resultant internal and external apoptosis, BBB and neuronal necrosis/neuroparenchymal damage [24,25,32,33]. This study clearly elucidated the role of SAP components, NF-κB, IL-1β, Casp 1, TNF-α and C3 expression in neuroimmunopathogenesis, establishing connections with other studies. These findings underscore that TE involves a highly complex and synchronized neuroimmunopathogenesis during the transition from acute systemic infection to early/mid-chronic and chronic phases.

There are notable studies, both in vivo and in vitro, that highlight the close relationship between SAP components and apoptosis. It has been demonstrated that SAP components significantly induce apoptosis in cerebrocortical cell cultures from rat brains [34,35] and after intrahippocampal administration [36]. Additionally, SAP components were found to bind to apoptotic cells in the early phase, emphasizing the close interaction between SAP components and apoptotic cells in vivo [37]. In our previous TE modeling, we revealed a pathological level of induced apoptosis in neurons, endothelial cells and glial cells at both 10 and 30 days post-infection [33]. We have clearly demonstrated that apoptosis is an extremely important factor in the immunopathogenesis of the observed neuropathology [33]. A 13.9-fold increase in SAP components was observed in the brain, which then rapidly decreased on days 20 and 30, reaching levels similar to those in the control groups. This study unequivocally illustrates that the 10th day after infection is a critical period. Therefore, it is apparent that SAP components, which increase in the brain during the 10-day period, play a role in neuropathogenesis by triggering apoptosis during this time frame. In the later periods (20 and 30 days post-infection), apoptosis is believed to be sustained by the factors described and discussed in our other studies.

Important targets of NF-κB include inducible nitric oxide synthase (iNOS) [38,39] and neuronal nitric oxide synthase (nNOS) [40,41], both of which can be highly neurotoxic, potentially triggering the production of reactive oxygen species (ROS). Nitric oxide (NO) reacts with superoxide to form the highly reactive peroxynitrite [42], known for its role as both an oxidant and a nitrating agent [42]. Peroxynitrite has been demonstrated to cause cellular damage, including DNA damage, and to activate apoptosis [43]. This process clearly indicates that NOS expression enhances ROS damage, paving the way for the formation of permanent pathology. It has also been shown that a mechanism involving TNF-α and the NF-κB pathway cooperates in the induction of oxidative stress (OS) in ALS. In brief, TNF-α-induced NF-κB activation has been shown to contribute to pathogenesis by increasing glutamate excitotoxicity on motor neurons [14]. In previous research, we demonstrated that high levels of endothelial nitric oxide synthase, iNOS and nNOS expression at the pathological level are involved in neuroimmunopathogenesis in TE models [25]. We have also shown that OS and its mediated consequences play an extremely important role in the development of TE neuropathology [32]. In this study, we show that NF-κB is elevated to pathological levels at 10, 20 and 30 days after infection. This clearly indicates NO-mediated neuropathology triggered by NF-κB. Additionally, we have demonstrated that the previously described OS, potentially mediated by triggered NO release, is actually induced by NF-κB, playing an active role in the neuroimmunopathogenesis of TE. In other words, the origin of OS and nitrosative stress is NF-κB targeting NOS. Moreover, considering that ROS activates Casp 1 through the pyrin domain-containing 3 protein of the Nod-like receptor family [44,45], another significant finding in this study is that IL-1β, produced by Casp 1 through the cleavage of inactive proIL-1β [17,18], is triggered in the context of OS.

Complement 3 (C3), an acute phase reactant, is a crucial component of the innate immune system. Together with other complement proteins, it plays a vital role in detecting and clearing potential pathogens [46,47]. The complement system serves as the first line of defense, rapidly mobilizing against invading pathogens [46]. In C3-deficient mice infected with *T. gondii*, uncontrolled parasite proliferation, acute mortality and a concurrent decrease in antibody production were observed [48]. The same study found that the complement system rendered mice resistant to acute infection but susceptible to chronic infection by limiting parasite proliferation in vivo [48]. This study demonstrates the importance of C3 in regulating parasite proliferation and antibody responses in vivo. *T. gondii* survives by establishing a critical balance in vivo through inactivation of the complement. We observed a significant increase in the C3 levels on the 20th and 30th days after infection in this study. Although there was an increase in C3 on the 10th day after infection, these increments were not significant compared to the control groups. C3 initiated a response to the infection but failed to reach the desired level at a critical time, such as 10 days after infection. This is likely due to *T. gondii*’s efforts to regulate C3 in its favor, causing partial suppression.

A study conducted in an experimental ischemic stroke model found that neuronal, vascular and oligodendrocyte damage caused by IL-1β overexpression in the central nervous system (CNS) was significantly increased [19]. Another study in human astrocytes demonstrated that IL-1β also contributed to the induction of interleukin-6 (IL-6), TNF-α and NOS [49,50]. In addition, in a study conducted in the TE model, IL-6 activity was found to be significantly higher in the brains of mice infected with *T. gondii* on the 10th, 20th and 30th days compared to healthy mice in the control group [51]. Furthermore, pro-inflammatory cytokines such as IL-1β and TNF-α have been identified as major contributors to synaptic and neuronal pathology in fatal neurodegenerative diseases such as EAE and MS [20,21]. Additionally, IL-1β and TNF-α have been implicated in the pathogenesis of PD [22]. In this study, we observed that TNF-α increased from day 10 post-infection and remained at high pathological levels, whereas IL-1β increased from day 20 to day 30 post-infection, which is a particularly intriguing finding. The fact that TNF-α elicits a rapid response at a critical time, such as the 10th day after infection, and that IL-1β follows in the subsequent days serves as a crucial determinant in neuroimmunopathogenesis. This raises the question of whether IL-1β plays a critical role in the persistence of latent infection. What is certain, however, is that the synergistic effects of both TNF-α and IL-1β in the development of neuropathology cannot be ignored.

*Toxoplasma gondii* infection has been reported to be associated with schizophrenia [52,53]. We previously explored this connection in detail, demonstrating that increased GMF expression, initially observed in TE, might play a crucial role in the pathogenesis of *T. gondii*-induced schizophrenia [24]. We highlighted the shared similarities in the pathogenesis of this neuropsychiatric disease with that of TE [24]. It was elucidated that members of the NF-κB family, along with all NF-κB activating receptors and many kinases, were upregulated to the pathological level in schizophrenia patients. This pathological activity was associated with immunological responses observed in the cortical region of schizophrenia patients [54]. In another study, pathologically higher levels of IL-1β and TNF-α serum, along with NF-κB activation, were found in schizophrenia patients compared to healthy individuals [55]. Notably, after 4 weeks of treatment with the neuroleptic risperidone, the serum levels of IL-1β in schizophrenia patients decreased significantly [55]. This and similar studies underscore the positive correlation between IL-1β, TNF-α and NF-κB activation, playing a pivotal role in the pathogenesis of the disease [54,55,56,57]. Based on the results of this study, it is suggested that *T. gondii*-mediated increased IL-1β, TNF-α expression and NF-κB activation in the brain contribute to the pathogenesis of schizophrenia. Therefore, this study conclusively demonstrates the involvement of the same pathways in the pathogenesis of *T. gondii*-induced schizophrenia. In other words, our findings are consistent with schizophrenia pathogenesis studies. In conclusion, this research reveals that, in the pathogenesis of schizophrenia developing after the infection of healthy individuals with *T. gondii*, the response to the *T. gondii*-mediated pathological levels of IL-1β, TNF-α expressions and NF-κB activation according to days provides an important avenue for the treatment or prevention of the disease.

The blood–brain barrier comprises a tightly connected network of capillary endothelial cells, pericytes and perivascular astrocytes. This structure serves to shield the brain from toxic substances and blood-borne pathogens while facilitating the removal of harmful compounds from the brain into the circulatory system [58,59]. As a result of IL-1β expression in the brain, both endothelial cells and astrocytes are affected, and there is an increase in the permeability of the BBB [13,60]. Furthermore, microglia-derived TNF-α has been shown to be the major factor exacerbating BBB impairment after ischemic stroke [61]. Furthermore, NF-κB has been shown to be associated with BBB deterioration after traumatic shock, and NF-κB inhibition was effective in protecting the BBB after traumatic shock [15]. With the presence of BBB efflux transport mechanisms protecting the brain from SAP penetration, SAP components do not enter the brain under physiological conditions [62]. Disruption of the integrity of the BBB and an increase in the concentration of the SAP components in the brain paves the way for the development of neuropathology [59]. We have previously shown that the integrity of the BBB is disrupted in TE, and consequently, neuropathology is exacerbated [25,32,33]. This study highlights the involvement of IL-1β, TNF-α and NF-κB in mediating BBB damage in TE. We observed a 13.9-fold increase in the concentration of the SAP components in brain tissue on the 10th day post-infection, providing concrete evidence of this impairment. These findings emphasize the importance of recognizing disruptions in the BBB integrity within the molecular context of TE neuropathogenesis. However, to ascertain the mechanisms underlying this damage and proactively mitigate it in the early phases, thus averting the progression of TE neuropathology, it is crucial to establish the day-dependent fluctuations in IL-1β, TNF-α and NF-κB.

## 5. Conclusions

This investigation reveals that the neuropathological alterations observed in toxoplasmic encephalitis (TE) are consistently influenced by dynamic changes in cytokines and molecular components, underscoring the intricate nature of neuropathogenesis. Within our TE model, we have scrutinized the roles and fluctuations of the SAP components, NF-κB, IL-1β, Casp 1, TNF-α and C3 gene expression at specific temporal junctures: day 10 (transiting from the acute to early chronic phase), day 20 (progressing to the early/moderate chronic phase) and day 30 (reaching the chronic phase). Particularly noteworthy is the significant contribution of pro-inflammatory cytokines IL-1β (manifesting from the 20th day post-infection) and TNF-α (evident from the 10th day post-infection) to the worsening of neuropathology. The discernment that SAP components play a pivotal role on day 10 post-infection, coupled with the sustained elevation of NF-κB throughout each infection period, underscores their substantial involvement in neuropathogenesis. These findings suggest that TE is in a continuous neuroimmunopathogenic continuum during the transition from acute systemic infection to the early/moderate and chronic stages. A critical reassessment of the treatment modalities, incorporating the dynamics observed during these pivotal days, emerges as imperative. It is of great significance to conduct comprehensive studies on this subject in order to ascertain which other components of the molecular mechanism of TE are involved in the acute phase proteins.

## Figures and Tables

**Table 1:** SAP components, NF-κB, IL-1β, Casp 1, TNF-α and C3 Taqman primer.

Gene	Primer Sequence (5′–3′)
SAP	Forward: GTCTTCACCAGCCTTCTTTCAGA Reverse: TCAGATTCTCTGGGGAACACAA
NFKB	Forward: CTATGATAGCAAAGCCCCGAATG Reverse: TCCTCCCCTCCCGTCACA
IL-1β	Forward: CAACCAACAAGTGATATTCTCCAT Reverse: GGGTGTGCCGTCTTTCATTA
CASP1	Forward: GCTGAGGTTGACATCACAGGCA Reverse: TGCTGTCAGAGGTCTTGTGCTC
TNF-α	Forward: AGCCAGGAGGGAGAACAGA Reverse: CAGTGAGTGAAAGGGACAGAAC
C3	Forward: CCATGTATTCCATCATTACTCCCA A Reverse: CGTGGGCCTCCAGTCAGA
β-actin	Forward: TCCTTCCTCTGATTAGCTGTCCTAA Reverse: TCCACATAATTTCCACCAACAAGT

**Table 2:** SAP, NF-κB, C3 mRNA gene expression results and statistical data for healthy controls and *T. gondii*-infected animals.

Animals	n	SAP		Between-Component Variance SAP	NF-κB		Between-Component Variance NF-κB	C3		Between-Component Variance C3
		Mean	SD		Mean	SD		Mean	SD	
Healthy control animals	6	8.062	0.863		14.146	0.642		10.954	0.859	
10th day after infection with *T. gondii*	6	114.037	2.616		33.713	1.085		13.019	2.24	
				2808.202			240.614			84.033
20th day after infection with *T. gondii*	6	7.765	0.754		51.602	1.321		25.982	0.774	
30th day after infection with *T. gondii*	6	8.31	1.066		38.375	1.088		29.317	1.009	

**Table 3:** IL-1β, Casp1, TNF-α mRNA gene expression results and statistical data for healthy controls and *T. gondii*-infected animals.

Animals	n	IL-1β		Between-Component Variance IL-1β	Casp1		Between-Component Variance Casp1	TNF-α		Between-Component Variance TNF-α
		Mean	SD		Mean	SD		Mean	SD	
Healthy control animals	6	3.949	0.932		12.204	0.6		4.204	0.669	
10th day after infection with *T. gondii*	6	4.278	0.884		36.06	0.704		21.972	1.399	
				626.159			217.289			563.168
20th day after infection with *T. gondii*	6	55.352	2.447		44.596	1.897		53.189	1.113	
30th day after infection with *T. gondii*	6	34.32	0.975		43.844	1.528		51.262	3.304	

## Data Availability

The original contributions presented in this study are included in the article. Further inquiries can be directed to the corresponding authors.
